# 

**Published:** 1898-03-26

**Authors:** 


					'gffi JiuariTAL. ~ "The standard ot nignest purity.??bancet. mahwi iwu, ib:b.
A
CADBURY'S ^ UT 1m CADBURYS
COCOA
NO DRUGS OR ALKALIES USED.
COCOA
,9
o
lftmcH hospi tal
'ULAsenw IVrsTFPV infirmary
No. 600. Vol. XXIII. [f^ewspfper!] SATURDAY,
March 26th, 1898.
-SnbBoriptionTema.] pBiICE TWOPENCE.

				

